# Identification of Evidence for Key Positive Psychological Constructs in Pediatric and Adolescent/Young Adult Patients with Cancer: A Scoping Review

**DOI:** 10.1089/jayao.2020.0184

**Published:** 2021-06-15

**Authors:** Cole Wayant, Jack Manquen, Hannah Wendelbo, Natalie Kerr, Matt Crow, Jon Goodell, Andrea C. Tricco, Jennifer W. Mack, Chan Hellman, Matt Vassar

**Affiliations:** ^1^ Department of Psychiatry and Behavioral Sciences and Oklahoma State University Center for Health Sciences, Tulsa, Oklahoma, USA.; ^2^ Department of Library Services, Oklahoma State University Center for Health Sciences, Tulsa, Oklahoma, USA.; ^3^ Li Ka Shing Knowledge Institute, St. Michael's Hospital, Unity Health Toronto, Toronto, Canada.; ^4^ Epidemiology Division, Dalla Lana School of Public Health, University of Toronto, Toronto, Canada.; ^5^ Division of Population Sciences, Department of Pediatric Oncology, Dana Farber Cancer Institute/Boston Children's Hospital, Boston, Massachusetts, USA.; ^6^ School of Social Work, University of Oklahoma, Norman, Oklahoma, USA.

**Keywords:** adolescent, young adult, oncology, psychology, psychosocial, well-being

## Abstract

***Introduction:*** Children and adolescents/young adults (AYAs) with cancer are a vulnerable population susceptible to numerous late effects, such as fatigue and depression, which may diminish their long-term psychological, physical, spiritual, and emotional health. A well-rounded understanding of how positive psychological constructs affect the quality of care and treatment outcomes is therefore warranted.

***Methods:*** We conducted a scoping review of 15 positive psychological constructs in children and AYAs with cancer. The primary research questions were (1) what is known about positive psychological constructs in children and AYAs with cancer; (2) what value is ascribed to these constructs by patients?

***Results:*** Two hundred seventy-six articles were included after database search and screening. These studies were mostly observational or qualitative and conducted in North America. Constructs were often poorly defined, and measurement tools used to gather data were wide ranging. Numerous factors were correlated with increased or decreased expression of certain constructs, but overall themes were difficult to identify. Similarly, patients often spoke of what increased or decreased expression of a construct, with less emphasis on what they implicitly value.

***Discussion:*** This scoping review found ample evidence for what increases or decreases expression of positive psychological constructs, but this evidence was observational and often conflicting. In the future, we recommend the development of a core set of psychological outcomes, with definitions and corresponding measurement tools. We further recommend an emphasis on randomized trials to more rigorously study how expression of constructs can be improved and what effect this has on the quality of life.

## Introduction

Children and adolescents/young adults (AYAs) with cancer are a vulnerable population, susceptible to numerous late effects, such as fatigue and depression, which may diminish their long-term psychological,^1,2^ physical,^1^ spiritual,^3^ and emotional^4^ health. Fostering positive psychological constructs—such as hope or optimism—during treatment and maintenance has been shown to correlate with improved rates of survival^5^ and quality of life^6^ in adult patients with cancer. To strengthen psychological care for children and AYAs with cancer, health care providers of pediatric and AYA cancer care must be equipped with robust evidence demonstrating the potential for psychological constructs to improve patient outcomes. However, a necessary first step before dedicating clinical resources to cultivating positive psychological constructs is to determine which constructs improve patient outcomes.

A significant portion of prior research has focused on negative psychological constructs, such as anxiety and depression, as predictors of poor health outcomes in cancer patients.^7,8^ The clinical importance of these studies is an onus on health care providers, such as doctors or nurses, to prevent, identify, and treat any negative psychological construct that arises in patients during cancer treatment. However, the mitigation or absence of negative psychological constructs does not equate to augmented positive psychological constructs, which are able to improve health outcomes in healthy and diseased populations. Moreover, positive psychological constructs may yet improve outcomes despite co-occurring negative constructs.^9^

A significant amount of research has been conducted with children and AYAs to investigate how psychological constructs function throughout cancer diagnosis, treatment, and follow-up (Evan and Zeltzer 2006).^10^ Much of this research is conflicting. For example, some studies have concluded that childhood cancer survivors are psychologically troubled (Hobbie et al. 2000; Meeske et al. 2001),^11,12^ whereas others conclude the opposite.^13^ Some of this research seems to have reached conclusive answers, such as how age may affect the ability of an AYA patient to cope (Jamison et al. 1986; Varni et al. 1994; Claflin and Barbarin 1991).^14–16^ However, given the breadth of the pediatric and AYA population and how family, developmental, and social factors may exert unique influences on individual patients, there is reason to believe that much is still unknown or unsolved. This becomes even more pressing when one considers the breadth of measurement instruments that have been developed, which may contribute to what seem to be conflicting results. Altogether, it is imperative that pediatric and AYA cancer research related to positive psychological constructs be collated in a systematic manner to identify key next steps to create efficient, patient-focused research objectives that can maximally improve psycho-oncological outcomes. A scoping review is an ideal method of accomplishing this task.

Scoping reviews systematically identify strengths and gaps in what is known about a topic. For example, a recent scoping review investigated practices or programs that promote AYA patient-centered communication.^17^ The authors of this scoping review found that only eight published articles were relevant to their review question and thus concluded that a significant gap exists in the literature regarding patient/provider communication in AYA oncology. The strength of a scoping review is to simultaneously answer a novel question (e.g., what is known on a topic) and generate new hypotheses that may be prioritized in future research. The chief aim of this scoping review is to map the existing literature regarding positive psychological constructs as they relate to health outcomes in children and AYAs with cancer. Beyond our immediate aim, the goal of this scoping review is to form the basis for a future psychometric systematic review and reliability generalization meta-analysis that will aim to identify the highest quality measurement instruments for positive constructs shown to improve health outcomes for children and AYAs with cancer.

## Methods

This scoping review is reported and conducted in accordance with the PRISMA Extension for Scoping Reviews (PRISMA-ScR) and the 2020 Joanna Briggs Institute (JBI) manual for scoping reviews.^18,19^ This review was registered through the Open Science Framework (OSF).^20^ Upon completion of this review, all data, metadata, and supplemental information will be publicly available through the OSF. This review was posted as a preprint to OSF Preprints.

### Literature search

The search strategy was created and optimized by C.W., M.V., and J.G. to identify all relevant literature regarding previously selected positive psychological constructs in children and AYAs with cancer (age 2–39). The positive constructs are shown in Table 1 and a search strategy was constructed for each, individually. The search strategies were adapted to the chosen databases in accordance with the JBI manual: PubMed (which includes Medline) and CINAHL, including all conference proceedings and dissertations. Two preprint servers—MedRxiv and PsyArXiv—were searched to identify additional studies that have not been published. J.G., a medical librarian, executed the final search.

**Table 1.** Number of Studies Per Construct with Construct Definitions

Construct	Definition	Number of articles	Article characteristics
Well-being	The positive components of psychological health that characterize individuals who feel good about life and function well^178^	96	Quantitative: 42 Qualitative: 34 Mixed Methods: 20 Proxy: 11
Personal growth	Positive psychological change that occurs following experience with adversity^179^	65	Quantitative: 36 Qualitative: 11 Mixed Methods: 18 Proxy: 5
Hope	The perceived capability to derive pathways to desired goals, and motivate oneself through agency thinking to use those pathways^180^	60	Quantitative: 25 Qualitative: 15 Mixed Methods: 20 Proxy: 5
Meaning in life	The cognizance of order, coherence, and purpose in one's existence, the pursuit and attainment of worthwhile goals, and an accompanying sense of fulfillment^181^	41	Quantitative: 24 Qualitative: 5 Mixed Methods: 12 Proxy: 3
Self-esteem	No *a priori* definition established, was included as incidental finding according to protocol	40	Quantitative: 24 Qualitative: 6 Mixed Methods: 10 Proxy: 5
Vitality	One's conscious experience of possessing energy and aliveness^182^	38	Quantitative: 18 Qualitative: 8 Mixed Methods: 12 Proxy: 7
Optimism	The belief that one's outcomes will be positive rather than negative^183^	36	Quantitative: 20 Qualitative: 4 Mixed Methods: 12 Proxy: 2
Resilience	No *a priori* definition established, was included as incidental finding according to protocol	29	Quantitative: 14 Qualitative: 4 Mixed Methods: 11 Proxy: 0
Gratitude	Generalized tendency to recognize and respond with grateful emotion to the roles of other people's benevolence in the positive experiences and outcomes that one obtains^184^	25	Quantitative: 14 Qualitative: 4 Mixed Methods: 7 Proxy: 1
Life satisfaction	*A* global assessment of a person's quality of life according to their chosen criteria^185^	23	Quantitative: 16 Qualitative: 1 Mixed Methods: 6 Proxy: 2
Self-acceptance	An individual's satisfaction or happiness with themself^186^	15	Quantitative: 4 Qualitative: 9 Mixed Methods: 2 Proxy: 1
Happiness	*A* positive emotional state that is most general and not restricted to any specific circumstances or events^187^	14	Quantitative: 8 Qualitative: 1 Mixed Methods: 5 Proxy: 1
Tranquility	*A* natural settling of thoughts and emotions, in which there is stability of attention, sensory clarity, and equanimity of affect and behavior^188^	9	Quantitative: 6 Qualitative: 2 Mixed Methods: 1 Proxy: 1
Perseverance	The ability to pursue one's goals to completion, even in the face of obstacles^189^	6	Quantitative: 2 Qualitative: 2 Mixed Methods: 2 Proxy: 0
Contentment	The perception that the present situation is enough and entire^190^	4	Quantitative: 1 Qualitative: 1 Mixed Methods: 2 Proxy: 1

### Research question

Based on the objective of this scoping review, the following research question was formulated: What is known from the published literature about positive psychological constructs and improvements in quality of life or survival among children and AYAs with cancer? The objective of this scoping review is to summarize and map the existing evidence about (1) which constructs, if any, children and AYAs consider important; (2) whether children/AYAs see value in cultivating positive psychological constructs during cancer treatment; and (3) whether cultivation of positive psychological factors is associated with improved quality of life or survival. In this study, we highlight key next steps that may include, but are not limited to: (1) recommendations for future research into specific psychological constructs; (2) recommendations to begin cultivating specific psychological constructs.

### Inclusion criteria

Broad inclusion criteria for this scoping review were conceptualized according to population, concept, and context. For this review, evidence reporting on prespecified positive psychological constructs in pediatric and AYA with previous or current cancer of any type were included. No date or language limitations were enforced. Included studies related to child/AYA beliefs about selected positive psychological constructs or data related to how the selected constructs correlate with improved quality of life or survival in children or AYAs. Interventional, observational, and qualitative study designs were eligible for inclusion, along with reviews of any kind.

Included evidence came from studies of children and AYAs currently being treated for cancer or survivors of childhood or AYA cancer. Studies that focus on parent or sibling perspectives were included if they focused on the child or AYA with cancer and his or her experiences, but were analyzed separately. No restrictions based on type of cancer were included in this analysis. If a study included patients outside the age range prespecified for this study (age 2–39), it was included only if it was confirmed that at least 50% of the included patients fell within the required age range.

### Screening

All screening of retrieved papers from the bibliographic database search was done in duplicate and blinding was maintained between screeners. Before screening, a pilot test of 50 randomly selected articles was completed. Greater than the prespecified 90% concordance between screeners was achieved after one pilot test. We used Rayyan^21^ to screen all references by title and abstract. The full text of studies was then examined to finalize the list of included studies using the same methods to ensure blinding.

### Data charting

Following the identification of included articles, we proceeded to data charting. Data charting, like article screening, was conducted in a dual, blinded fashion using an extraction form optimized for use in Google Forms. A pilot test was conducted to optimize the extraction form, similar to the methods described in the Screening section. Prespecified information to be gathered on positive psychological constructs included qualitative data, quantitative data, or both, depending on the design of the included studies. The purpose of this phase of the scoping review was to chart the strengths and gaps in our knowledge of positive psychological factors on health outcomes in children and AYAs.

### Synthesis

Fifteen positive psychological constructs were selected based on previously published literature related to cancer, diabetes, and cardiovascular outcomes.^6,22,23^ Data were cataloged according to the 15 chosen constructs. New constructs, beyond those initially searched that were identified, were included as novel constructs and analyzed separately. Included studies and their constructs were judged against the definitions for included constructs seen in Table 1. If an included study did not define a construct, we did our best to judge whether included data were relevant. For example, if a patient expressed appreciation for their ability to complete small, day-to-day tasks, we would categorize this as “gratitude,” even if the study did not offer any definition of gratitude.

Due to the nature of this scoping review, there were no statistical analyses planned. Included data were reported using simple thematic categorization. Proposed themes included favorable, unfavorable, or indifferent patient views or quantitative data regarding specific positive constructs in the context of cancer treatment. All data were charted and recommendations for future research are made with as much specificity as possible, based on age at diagnosis, race, gender, type of cancer, and other socioeconomic factors identified. For age at diagnosis, we attempted to categorize data according to three distinct groups of pediatric/AYA cancer patients/survivors: those <15 years of age, between 15-21 years of age, and >21 years of age.

## Results

### Characteristics of included studies

**FIG. 1.**
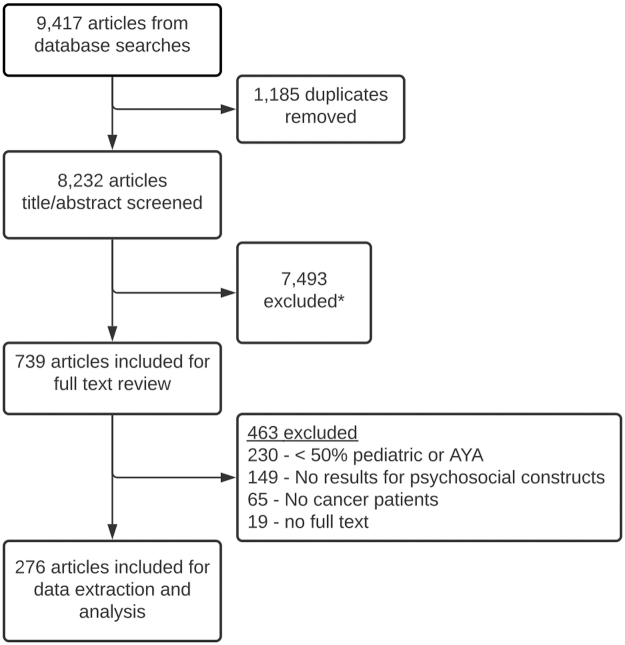
Flow diagram of included and excluded studies. *Reasons for inclusion include <50% pediatric or AYA, no results for psychological constructs, no cancer patients. AYA, adolescent and young adult.

**FIG. 2.**
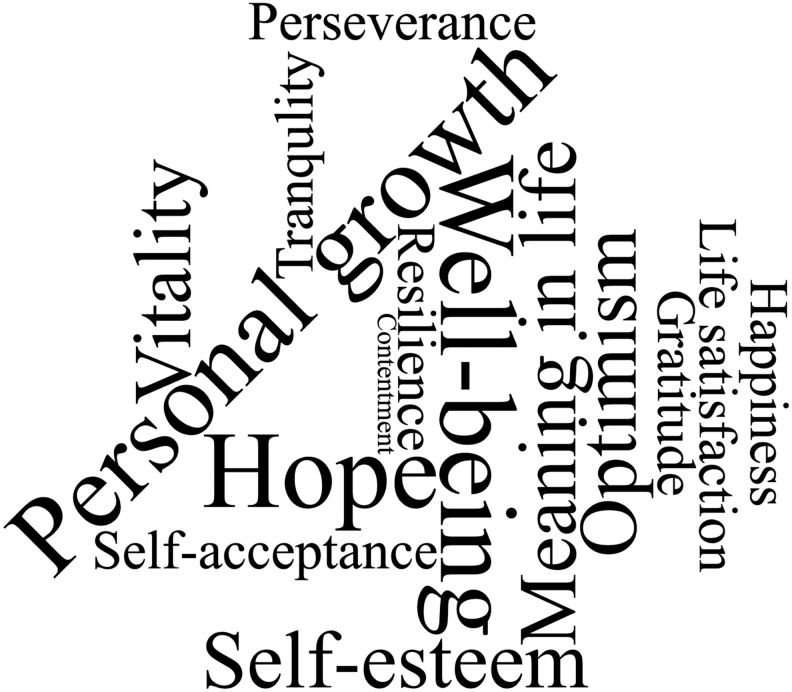
Word cloud of constructs and their frequency in included articles.

Overall, 9417 articles were returned from our database searches. After removing duplicates (n -1185), 8232 articles were screened for inclusion. After applying inclusion and exclusion criteria, 739 articles were eligible for full-text review. After a full-text review, 276 were included as our final sample. A flow diagram of all inclusions and exclusions is shown in Figure 1. No data for the constructs of cheerfulness and enthusiasm were found in our final sample; however, two new constructs were identified: resilience and self-esteem (Fig. 2). Eighteen potentially relevant studies were identified from preprint servers, but zero met inclusion criteria.

Included studies had a median sample size of 60 (IQR 17–171.75) and included patients from 38 countries, most often the United States (*n* = 114, 41.3%), Canada (*n* = 27, 9.8%), and Sweden (*n* = 15, 5.4%) (Table 2). These included studies were mostly observational (*n* = 91, 32.9%), interviews (*n* = 74, 26.8%), mixed-methods studies (*n* = 47, 17.0%), or narrative reviews (*n* = 25, 9.1%). Few randomized-controlled trials (*n* = 11, 4.0%) and systematic reviews (*n* = 15, 5.4%) were included. Categorizing studies by age group was challenging due to individual study reporting. The majority of included studies included patients whose ages spanned more than one prespecified group (*n* = 158, 57.2%). Overall, 57 (20.7%) studies were restricted to patients less than 15 years of age, 16 (5.8%) to 15–21-year olds, and 21 (7.6%) to 22–39-year olds. Few studies evaluated a single cancer (*n* = 44, 15.9%). Overall, studies most often included patients with leukemia (*n* = 198, 71.7%), lymphomas (*n* = 64.5%), and central nervous system tumors (*n* = 120, 43.5%). A significant number of studies did not, in part or in whole, specify which tumors they include (*n* = 107, 38.8%). Few studies included proxy reports (*n* = 28, 10.1%). All included constructs were measured by at least 2 different measurement tools, with growth, well-being, optimism, resilience, and self-esteem each being measured by more than 10.

**Table 2.** Summary of Study and Included Patient Characteristics

Included cancers (most common)	
Leukemia	
Unspecified type	113 (40.94%)
Acute lymphoblastic	58 (21.01%)
Acute myeloid	20 (7.25%)
Lymphoma	
Unspecified type	86 (31.16%)
Hodgkin's	49 (17.75%)
non-Hodgkin's	40 (14.49%)
Sarcoma	
Soft tissue	58 (21.01%)
Bone	45 (16.30%)
Ewing	24 (8.70%)
Central nervous system	120 (43.48%)
Other (not specified)	107 (38.77%)
Germ cell	69 (25.0%)
Patient age groups^*^	
< 15 years old	57 (20.65)
15–21 years old	16 (5.80%)
22–39 years old	42 (15.22%)
Ages span more than 1 group	158 (57.25%)
Proxy reports only, ages not specified	3 (1.09%)
Study designs	
Observational	91 (32.9%)
Interview	74 (26.81%)
Mixed-Methods	47 (17.0%)
Review	25 (9.06%)
Systematic review	15 (5.43%)
Randomized controlled trial	11 (3.99%)
Nonrandomized trials	6 (2.17%)
Case study	2 (0.72%)
Post-hoc analysis of trial	2 (0.72%)
Predictive model	1 (0.36%)
Psychometric validation study	1 (0.36%)
Conference presentation	1 (0.36%)
Location of patients^**^	
USA	114
Canada	27
Sweden	15
Australia	12
Netherlands	12
Italy	10
United Kingdom	10

^*^ Grouped according to these criteria due to variations between studies. ^**^Counts greater than 10, some studies included patients from more than 1 country.

### Psychological constructs

*Well-being* was studied by 96 (34.7%) included articles. There was conflicting evidence about whether patient well-being was better,^24,25^ worse,^26–28^ or no different from healthy controls.^29–31^ Most often, well-being was correlated with increased social support.^32–35^ Physical activity^36,37^ and art making^38,39^ also correlated with increased well-being. Health care workers played an important role in patient well-being through good communication,^31^ comprehensive care,^40^ and encouraging appropriate expectations of treatment.^41^

Negative body image,^33,42,43^ anxiety, depression,^44,45^ and fatigue^46,47^ decreased patient well-being. Moreover, treatment side effects, especially pain,^48^ correlated with lower well-being. Last, ample evidence, from primary and proxy reports, indicated that as time passed from diagnosis, well-being decreased.^49–51^

Personal growth was described by 65 (23.6%) articles in our sample. The most significant factor contributing to personal growth was the cancer experience itself (37/65, 56.9%). Some specific areas of growth mentioned were improved self-reflection,^52^ clearer life purpose,^53^ a positive new identity,^54,55^ overall maturation,^56^ and increased empathy.^57^ Faith in God and spiritual struggles played a significant role in patient personal growth,^58,59^ mostly by allowing patients to adapt to or comprehend their disease. Social support systems helped promote growth in patients with cancer.^60,61^ Last, inner resources of patients, including self-esteem,^62^ optimism,^63^ and self-affirmation,^64^ were shown to improve growth in patients with cancer.

Survivors of cancer described how body image changes and perceived changes to their social reputation among peers^65^ were factors which decreased personal growth. Depression^66^ and stress^67^ also played a role.

Hope in cancer patients was reported by 60 (21.7%) articles. One study identified three main objects of hope for patients with cancer^68^: breakthrough treatments, cures,^69,70^ and future children or family.^71–74^ Hope was shown to predict resilience,^75,76^ mitigate future distress,^77^ and aid recovery.^78^ Hope was important for patients to derive meaning and cope with their illness.^79–81^

Increased hope was correlated with positive rumination,^82^ humor, belief in God,^83^ self esteem,^80^ optimism,^84^ and peer/family support.^85,86^ Other factors were related to health care providers, with nurses being especially important.^87,88^ As it relates to physicians, studies highlighted the importance of honest, complete communication with patients about prognosis.^83,89,90^

Factors which correlated with decreased hope included anxiety, depression,^82^ infertility,^91^ and dissatisfaction with oncologist communication. One father described how his son's oncologist “undid some of our work on hope” by disclosing his son's prognosis, against his son's wishes.^92^

Meaning in life for patients with cancer was discussed by 41 (14.9%) articles. Cancer facilitated meaning discovery through refined career goals,^52,93^ self reflection,^73^ new religious perspectives,^94^ and giving patients a purpose.^95^ One study suggested that patients may find meaning in cancer by: attempting to define the disease, viewing cancer as a ‘‘divine test’’, or as a catalyst for positive self-reconstruction.^96^ Intrinsic factors, such as self-esteem,^44^ religious faith,^97^ spiritual well-being,^98^ and positive coping strategies,^99^ correlated with meaning discovery. Interventions that may improve meaning discovery include increasing social support,^98^ legacy making through story or art,^100^ increased benevolence toward others,^64^ and certain meaning-centered programs.^101^ Finally, there is evidence that fulfilling employment, meeting education goals,^102^ and accomplishing tasks^103^ may improve meaning discovery in patients with cancer.

Factors that interfered with meaning discovery included anxiety, depression,^104^ negative emotions,^105^ and a sense that there is little time left to live.^106^

Self-esteem was described by 40 (14.5%) articles to be increased by peer engagement, hope, academic success, and physical activity.^34,80,84^ Patients commonly attributed their self-esteem to having and surviving cancer,^107–109^ as well as relationships with health care providers.^110^ Factors that decreased self-esteem included poor body image,^29,73,111^ sexual dysfunction,^112^ physical impairment,^113^ and fatigue.^29^

Included articles did not agree with respect to whether patients with cancer have higher self-esteem than healthy controls. Some found higher,^25,113^ lower,^108,114^ or equal^28,115^ self-esteem in patients.

Vitality among patients with cancer was reported in 38 (13.8%) of included studies, most often as a component of overall quality of life or well-being. Few factors were reported that increased vitality, including marriage,^116^ yoga,^117^ and stronger overall mental or physical health.^118,119^ There were conflicting results for whether time since diagnosis improves vitality.^120–123^

Decreased vitality was seen in patients with lower income, a longer disease course, a longer hospital stay, and sexual dysfunction.^124,125^ More conflicting evidence about vitality was found for patients relative to healthy controls; five studies observed no difference,^125–129^ three observed lower vitality,^130–132^ and three observed higher vitality.^133–135^ Demographic characteristics followed a similar trend. For example, one study found older patients have more vitality than younger,^121^ whereas three studies showed the opposite.^125,131,136^

Optimism was a focus of 36 (13.0%) included articles. Multiple studies reported that optimism was increased during the cancer experience^94,137–139^ and that optimism improved a patient's ability to cope with cancer.^71,102,140^ Family and peer support were important for increasing optimism.^141^ Other factors such as religious faith,^59^ hope,^142^ posttraumatic growth,^66^ and strong mental health^143,144^ were correlated with higher levels of optimism.

Factors that decreased optimism were less often reported. Proxy reports indicate that pessimism^145^ and a diagnosis of brain cancer^56^ correlated with lower levels of optimism in patients with cancer.

*Resilience* was frequently discussed (29/276, 10.5%), despite not being included in our original search. The cancer experience itself induced resilience,^34,108,146^ similar to engaging in positive coping strategies,^42,66,75^ such as setting and maintaining future goals.^78^ When patients felt any form of connection or belonging, resilience was reported to increase.^66,147^ Religious faith may be one form of connection or belonging.^59^ Similar to other constructs, anxiety, distress, pessimism, and a feeling of not knowing what to expect decreased resiliency.^66,78^

Gratitude was evaluated by 25 (9.1%) included articles. No factors were found that decreased gratitude. The three predominant factors that increased gratitude were: having and surviving cancer,^55,57,148^ peer support,^103,149^ and a strong relationship with their health care provider.^110,150^ Cancer was described as helping patients appreciate the small things in life.^57^ Family selflessness was important for cancer patients, with one cancer patient describing how newfound fatherly affection made them the most grateful.^103^ Moreover, when health care providers made time for patients and treated them as individuals, gratitude increased.^110,150^ Other factors that were associated with increased gratitude were religious faith,^151^ the possibility of having a future family,^71^ and understanding the finiteness of life.^91^ Interestingly, patients said that their gratitude as a child improved health care follow-up in adulthood through increased personal responsibility.^152^

Satisfaction with life or circumstance was described by 23 (8.3%) articles. Patients described how finding ways to grow as a person,^153^ focus on just the present,^64^ engage with friends and family,^154,155^ and maintain a positive affect or outlook^156^ were factors that increased life satisfaction. Improved social skills and perceptions of their health correlated with improved life satisfaction.^153^ However, factors that eroded life satisfaction ranged from depression to anxiety to somatic late effects of cancer to longer treatment duration.^157^ Sexual dysfunction and disfigurement from treatment contributed to lower life satisfaction.^124,158^

*Self-acceptance* was discussed by 15 (5.4%) included articles. None focused on mechanisms or factors that decrease patient self-acceptance. In a similar fashion to other constructs, the cancer experience was most often shown to increase self-acceptance through increased optimism, heightened existential awareness, and more positive self-beliefs.^34,94^

Anticipatory guidance about possible physical changes during cancer treatment increased female patient self-acceptance,^33^ as did peer engagement^159^ and a focus on social–emotional well-being.^44^ When cancer patients were reminded or shown that they are capable of accomplishing tasks like peers without cancer, their self-acceptance increased.^54,103^ Mind–body exercises, like yoga and tai-chi, improved self-acceptance, and, according to patients, this occurred by demonstrating their body's physical capabilities were intact.^33,117^

*Happiness* was studied by 14 (5.1%) included articles. The factors most commonly attributed to increased happiness was the cancer experience and the suffering it caused,^94^ the relief of completing treatment,^160^ and gratitude for suffering less than expected.^141^ In many cases, it was implied that patient happiness was relative to others, and may not represent increased happiness from baseline before cancer. A randomized trial showed that guided imagination and drawing–storytelling increased patient happiness.^161^ A qualitative study indicated that displaying patient artwork made the hospital feel less “clinical”.^39^ If patients were disfigured^158^ or subjected to a more intense treatment regimen,^162^ their happiness was reported to decline.

*Tranquility* was mentioned by 9 (3.3%) articles included in this study. Similar to other constructs, the cancer experiences increased tranquility, although through patients experiencing a nearness to death and suffering.^96^ Interventions that were found to improve tranquility ranged from advanced care planning^163^ to high-quality communication from health care providers^164^ to prayer and religious engagement.^165^ On the contrary, fatigue, depression, and anxiety all eroded tranquility among cancer patients.^46,164^ There was also evidence from one observational study that cancer survivors were less tranquil over time than healthy controls.^166^

*Perseverance* was discussed in 6 (2.2%) included articles. Factors found to increase perseverance of pediatric and AYA cancer patients were: the cancer experience,^34,167^ hope for a future cure,^168^ and relationships with oncology nurses.^87^ The cancer experience was described as giving patients an “unknown strength” by patients,^167^ whereas hope for a cure allowed patients to persevere despite treatment side effects. Finally, perseverance was described as an active choice in the face of disease progression.^141^

*Contentment* was discussed in 4 (1.4%) articles in our final sample. Overall, four unique factors, each supported by a single study, were found to increase contentment in pediatric and AYA cancer patients: home cancer treatments, increased self-esteem, gratitude for having fewer negative cancer experiences, and surviving cancer.^64,107,169^ Moreover, receiving a diagnosis of cancer was found to decrease contentment in a positive manner, by driving patients to see fulfillment and meaning in their life.^170^

## Discussion

This scoping review of key positive psychological constructs in pediatric and AYA patients with cancer found a significant amount of observational research and mixed methods research, with less focus on patient interviews, and little focus on interventions. The result is that our study shows what may correlate with increased or decreased expression of included positive constructs, but is hindered in its ability to identify key mechanisms to improve the psychological care of pediatric and AYA patients with cancer. We did not find any studies correlating improved psychological care with survival benefits. Nonetheless, our study is able to provide key recommendations for future research in pediatric and AYA patients with cancer, which include the identification of a core set of positive psychological constructs, use of standardized measurement tools, and the testing of interventions with randomized trials (Table 3).

**Table 3.** Proposed Nonpharmacological Interventions That May Be Tested in Future Clinical Trials

Intervention	Construct(s) to which it may apply
Home chemotherapy	Well-being, contentment
Physical activity	Well-being, satisfaction, self-esteem, vitality, self-acceptance
Art-making	Well-being, happiness, meaning
Social or clinical support mechanisms	Well-being, growth, hope, meaning, optimism, gratitude
Advanced care planning	Tranquility
Communication with patients	Well-being, hope, tranquility

To begin, we identified the included constructs by reviewing the pediatric cancer, diabetes, and cardiovascular literature.^6,22,23^ We were unable to identify a core set of psychological constructs that play the largest role in oncology care for pediatric and AYA patients. Moreover, we were hindered in our ability to determine if the studies we included used compatible definitions for constructs. Many included studies did not define the construct they were studying, making it difficult for us to assess their results. An example has to do with the construct “vitality”. It was often unclear if authors were assessing patients for vitality in the sense of physical capacity to perform activities, or in the sense of mental energy and aliveness. Only the latter is a psychological construct. A core set of psychological constructs would help resolve this issue by standardizing the name and definition of psychological outcomes.^171^ Core outcome sets have been used across the medical literature and represent the minimum set of outcomes that should be reported in a scientific discipline.^172,173^ These outcomes are chosen by patients, caregivers, physicians, and other stakeholders using robust, Delphi methodology.^174^ For children and AYAs with cancer, this core outcome set may vary if the patient is at the end of life or palliative treatment setting. In the present case, perhaps highly related constructs, like contentment and tranquility can be combined and standardized so that the literature on these constructs is more unified and powerful.

Next, as a continuation of identifying a core set of psychological constructs, we recommend that measurement tools be studied with more scrutiny, since the tools used in our study were wide ranging and applied to diverse ranges of patients. For all constructs, at least two measurement instruments were used by included studies, with five constructs being measured by more than 10 unique instruments or versions of instruments. The goal of this study was not to test the robustness of measurement tools; however, we suspect that one measurement tool may not be relevant to both pediatric and AYA populations. As it stands, the pediatric and AYA population is one of the most diverse in all clinical oncology^175,176^; thus, more precision is required when planning studies, choosing measurement tools, and gathering data.

Last, our study showed that multiple factors correlated with increased expression of included positive psychological constructs, but there is evidence that this data may not be robust. For example, patients with cancer had higher, lower, or no different expressions of certain constructs when compared with healthy controls. Nonetheless, there is a framework for interventions being tested to improve expression of positive constructs. The PRISM intervention^177^—a skill-based, early palliative care intervention targeting stress management, goal setting, cognitive reframing, and meaning making—was tested in a randomized trial and shown to improve expression of resilience in the primary analysis, as well as hope, optimism, well-being, and personal growth in a post-hoc analysis. Moreover, a recent review of psychological interventions showed that the vast majority of those studied found favorable results.^146^ Therefore, while our call for increased attention to randomized testing of psychological interventions in the pediatric and AYA patient population is not new, our study shows yet again that more decisive research is needed to improve the psychological care of patients with cancer.

This study is limited by factors previously discussed: lack of definition of included constructs and conflicting data, which occasionally hindered reaching consensus. Nonetheless, our scoping review searched the medical and psychological literature broadly, and was able to make key recommendations to improve the psycho-oncology literature going forward as it pertains to pediatric and AYA patients.

In conclusion, this study of 15 positive psychological constructs in pediatric and AYA oncology found that much of the literature is observational or qualitative, with less reliance on randomized trials. Moreover, the included studies used a diverse set of measurement tools, and it is unclear whether these tools are appropriate for all participants. In the future, we recommend the study of psychosocial constructs in the context of clinical trials (with disease severity in mind) and the development of a core set of psychological outcomes and measurement instruments.
